# “Two for One”, Novel Dual Left Anterior Descending Artery (LAD) Variant: Type XIII

**DOI:** 10.7759/cureus.14717

**Published:** 2021-04-27

**Authors:** James R Pellegrini, Rezwan Munshi, Alejandro Alvarez Betancourt, Billal Tokhi, Amgad N Makaryus

**Affiliations:** 1 Internal Medicine, Nassau University Medical Center, East Meadow, USA; 2 Cardiology, Nassau University Medical Center, East Meadow, USA; 3 Cardiology, Northwell Health, Manhasset, USA

**Keywords:** ct coronary angiography, dual lad, acs, atypical chest pain, anatomical variant, lad variant, cardiac anatomy

## Abstract

Dual left anterior descending artery (LAD) is a rare phenomenon that occurs in less than one percent of the population. To date, 12 variants have been identified. Proper identification of coronary vessels is crucial in emergent situations that require prompt action, such as percutaneous coronary intervention (PCI). We propose that our case highlights a novel 13th (type XIII) variant. We present the case of a 57-year-old African American woman with a past medical history of hypertension, glaucoma, cerebral vascular accident, dyslipidemia who presented to the ED complaining of atypical chest pain for one day duration. Electrocardiography showed normal sinus rhythm at 60 beats per minute (bpm), normal axis, normal intervals, no acute ischemic changes, and an isolated T wave inversion in DIII. Cardiac markers were within normal limits. The patient was started on aspirin 81mg, atorvastatin 40mg, and restarted on amlodipine 5mg. Echocardiography showed a left ventricular ejection fraction (LVEF): 65%, normal right ventricular size and systolic function, mild mitral valve regurgitation, and mild aortic regurgitation. Computed tomographic (CT) angiography showed a novel subtype of dual LAD, the left circumflex and right coronary arteries were patent. The patient was discharged once stabilized and advised to follow up with cardiology. Dual LAD describes a rare anatomic variant in which two coronary branches, known as short and long LAD arteries, supply the territory normally supplied by the solitary LAD artery. To date, 12 variants of dual LAD, classified by origin and course of the short and long LAD arteries, have been described in the literature. To the best of our knowledge, the current case describes a novel subtype of dual LAD, variant XIII. The LAD originates as usual from the left main coronary artery (LMCA) and initially runs in the anterior interventricular groove for a short course before bifurcating into two long LADs which both leave the interventricular groove and course out to the apex. One of the vessels courses laterally and the other courses medially of the interventricular groove. It is pertinent to identify the coronary vessels accurately before certain interventions are taken. Acknowledgement of this phenomenon can help guide accurate management in the future for patients with this condition.

## Introduction

Dual left anterior descending artery (LAD) is a rare phenomenon that occurs in less than one percent of the population [1]. To date, 12 variants have been identified [2]. Proper identification of coronary vessels is crucial in emergent situations that require prompt action, such as percutaneous coronary intervention (PCI). We propose that our case highlights a novel 13th (type XIII) variant. We present the case of a middle-aged black female who presented to the ED complaining of substernal non-radiating atypical chest pain after witnessing an altercation. Upon admission cardiac imaging revealed a novel subtype of dual LAD. The patient was discharged once stabilized and advised to follow up with cardiology. It is pertinent to identify the coronary vessels accurately before certain interventions are taken. Acknowledgement of this phenomenon can help guide accurate management in the future for patients with this condition.

## Case presentation

The patient is a 57-year-old African American woman with a past medical history of hypertension, glaucoma, cerebral vascular accident (two times, once in 2014 and once in 2016, with no residual deficits), and dyslipidemia who presented to the emergency department (ED) complaining of atypical chest pain for one day’s duration. The patient stated that her chest pain started after she witnessed an altercation. She mentioned the pain was substernal, tight/pressure-like in nature, non-radiating, worse with anxiety, and 6/10 in intensity. Her new pain came in the setting of vertigo (for the past four months), lightheadedness, and difficulty ambulating. She denied any alleviating symptoms and the rest of the review of systems was non-pertinent.

**Figure 1:**
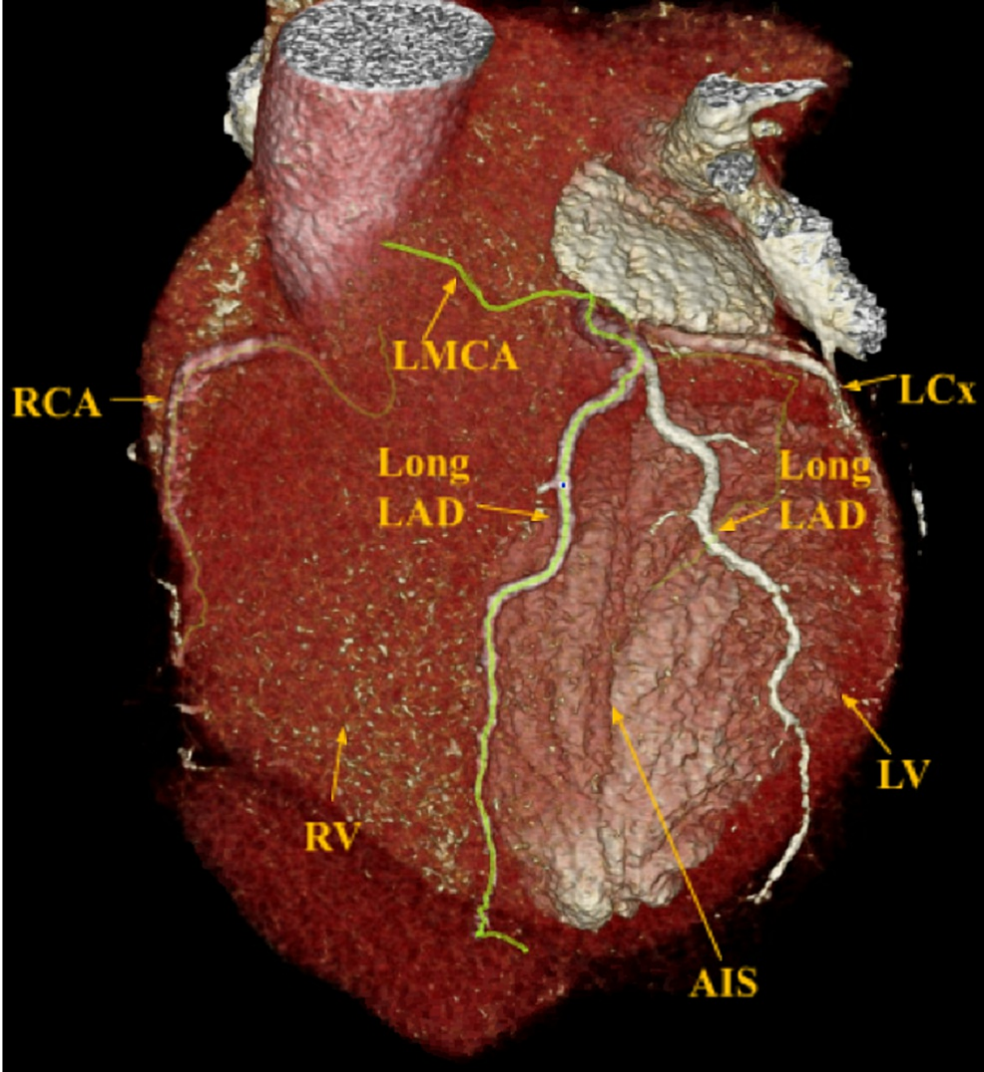
CCT showing two long LADs, one coursing laterally and the other coursing medial to the AIS CCT: Coronary computed tomography; LAD: Left anterior descending artery; AIS: Anterior interventricular sulcus.

**Figure 2:**
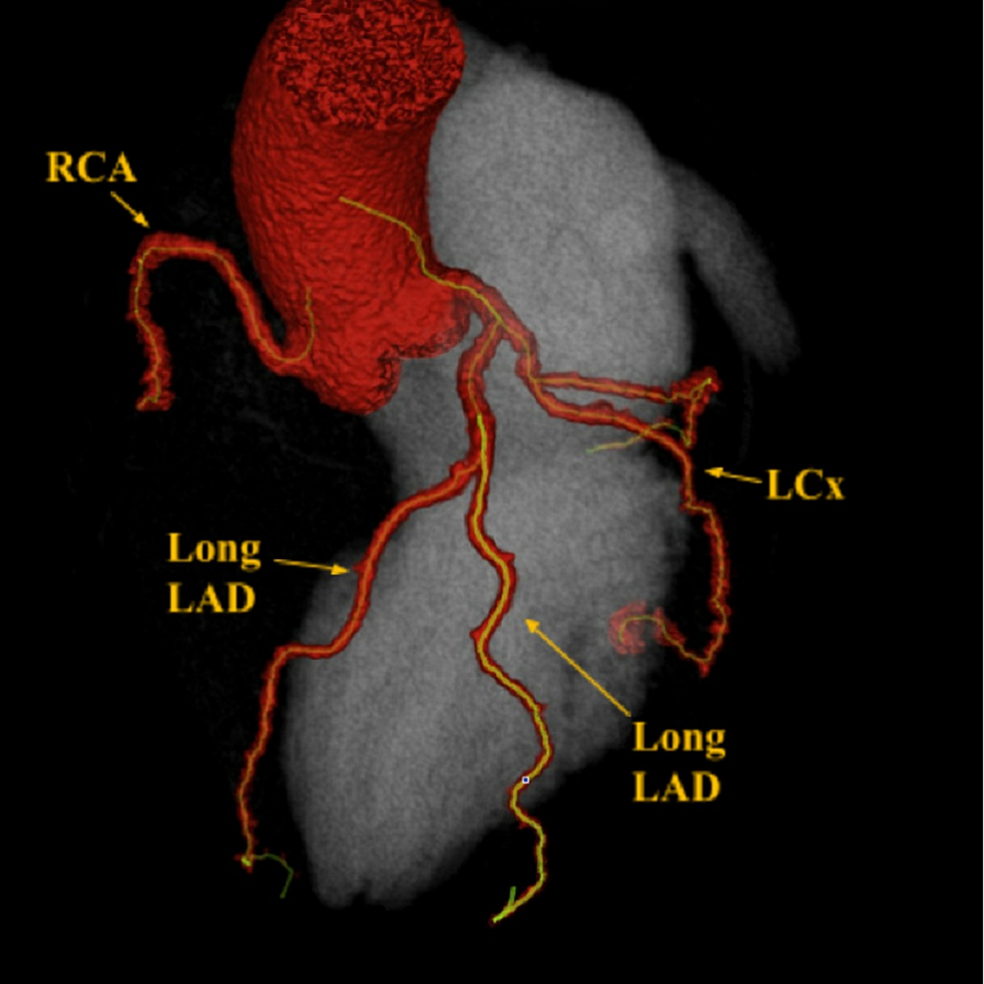
CCT showing two long LADs CCT: Coronary computed tomography; LAD: Left anterior descending artery.

Physical exam was unremarkable, except for mild tenderness to palpation of the chest wall and anxiety. An electrocardiography (ECG) was performed and showed normal sinus rhythm at 60 beats per minute (bpm), normal axis, normal intervals, no acute ischemic changes, and an isolated T wave inversion in lead 3. Vitals in the ED were normal except for elevated blood pressure at 170/84. Cardiac markers were within normal limits. Echocardiography showed a left ventricular ejection fraction of 65%, normal right ventricular size and systolic function, mild mitral valve regurgitation, and mild aortic regurgitation. Coronary computed tomography (CCT) (Figures 1, 2) was performed to evaluate coronary artery anatomy and assess plaque burden.

## Discussion

Differential diagnosis included acute coronary syndrome (ACS), musculoskeletal pain, and anxiety-induced chest pain. ACS was ruled out after cardiac markers were negative twice and ECG had no acute ischemic changes. Incidentally, the CCT results showed a left anterior descending artery (LAD) that originates from the left main coronary artery (LMCA) and initially runs in the anterior interventricular sulcus (AIS) for a short course before bifurcating into two long LADs which both leave the AIS and course out to supply the apex. While it may be considered that the second long LAD may represent a large first diagonal branch; that would assume that the patient has an LAD that resides in the expected anatomic position in the anterior interventricular groove, but in fact it does not, and instead another Long LAD, that runs medially to supply the septum and medial structures, is noted and does not run in the anterior interventricular groove [3]. One of the vessels was noted to course laterally and the other coursed medially of the AIS. The left circumflex (LCx) and right coronary arteries (RCA) were patent (Figures 1, 2).

The patient was continued on her home medications for hypertension - amlodipine 5mg oral daily. She was discharged once stabilized and advised to follow up as an outpatient. While dual LAD is rare (Sidhu and Wander found 0.68% prevalence for any type of dual LAD) and presumed benign, it presents various diagnoses and treatment challenges of which practitioners must be aware [4-6]. Prerequisite to successful surgical intervention, like revascularization, is the precise knowledge of the anatomical features to be evaluated. For example, if both the short and long LAD arteries are significantly stenosed in a patient, revascularization of both vessels may be necessary to restore blood supply to the septum and anterior wall. Additionally, as CCT has become the noninvasive imaging modality of choice for the diagnosis of cardiovascular coronary pathology, it is particularly imperative that clinicians be aware of these variants to allow for precise evaluation and management.

Typically, the LMCA gives rise to the LAD artery, which descends through the epicardial fat of the AIS. It then proceeds to give off septal and diagonal branches which supply the anteroseptal and anteroapical regions of the heart [1]. Dual LAD describes a rare anatomic variant in which two coronary branches, known as the “short” and “long” LAD arteries, supply the territory normally supplied by the solitary LAD artery.

Dual LAD was first reviewed and classified by Spindola-Franco et al. in 1983 when just three variants had been discovered [2]. To date, 12 variants of dual LAD, classified by origin and course of the short and long LAD arteries, have been described in the literature. Retrospective analyses have revealed that Type I dual LAD (in which the short LAD artery originates from the LAD proper and terminates in the AIS, while the long LAD artery originates from the LAD proper and descends to the left of the AIS before entering the AIS more distally) is the most common variant. Current classification of dual LAD can be seen in Table 1.

**Table 1:** Classification of dual LAD variants AIS: Anterior Interventricular Sulcus, LAD: Left Anterior Descending Artery, LMCA: Left Main Coronary Artery, LCS: Left Coronary Sinus, LV: Left Ventricle, RCA: Right Coronary Artery, RCS: Right Coronary Sinus, RVOT: Right Ventricle Outflow Tract, RV: Right Ventricle

Type	Short LAD Origin	Long LAD Origin	Short LAD Course	Long LAD Course
I	LAD Proper	LAD Proper	Originates from the LAD proper and terminates in the proximal AIS	Descends on LV side of the proximal AIS and enters the distal anterior interventricular sulcus
II	LAD Proper	LAD Proper	Originates from the LAD proper and terminates in the proximal AIS	Descends on the RV side of the proximal AIS and reenters the distal AIS
III	LAD Proper	LAD Proper	Originates from the LAD proper and terminates in the proximal AIS	Courses through the intramyocardial septum proximally, and may emerge in the distal AIS or terminate in the apical septum
IV	LMCA	RCA	Originates from the LMCA and terminates in the proximal AIS	Courses along an anomalous prepulmonic course anterior to the right ventricular outflow tract (RVOT) and reenters the distal AIS
V	LCS	RCS	Originates from the left coronary sinus (LCS) and terminates in the proximal AIS	Courses along an anomalous intramyocardial course within the septal crest, emerges epicardially, to enter the distal AIS
VI	LMCA	RCA	Originates from the LMCA and terminates in the proximal AIS	Courses between the RVOT and the aortic root and emerges in distal AIS
VII	LAD Proper	LAD Proper	Originates from the LAD proper and terminates in the proximal AIS	Courses along the LV side of the proximal AIS and reenters the distal AIS
VIII	LMCA	Mid-RCA	Originates from the LMCA and terminates in the proximal AIS	Courses along the inferior wall of the RV and traverses the apex to terminate in the distal AIS
IX	LAD Proper	LAD Proper	Originates from the LAD proper and terminates in the proximal AIS	Courses along the LV side of the AIS, enters the distal AIS and terminates before the apex
X	LMCA	RCS	Originates from the LMCA and terminates in the proximal AIS	Courses along an anomalous prepulmonic course anterior to the RVOT and reenters the distal AIS
XI	RCS	RCS	Originates from the RCS, takes an intramyocardial course within proximal septum and emerges in the proximal AIS	Courses along an anomalous prepulmonic course anterior to the RVOT and reenters the distal AIS
XII	LMCA	RCS	Originates from LMCA that originates in the RCS and terminates in the proximal AIS	Courses anterior to the main pulmonary artery and terminates in the distal AIS
XIII	No Short LAD	LAD Proper LAD Proper	Not applicable	Two long LADs which both leave the AIS and course out to the apex. One of the vessels courses laterally and the other courses medially of the AIS

## Conclusions

To the best of our knowledge, the current case describes a novel subtype of dual LAD, which we propose to name variant XIII. In theory, mortality associated with LAD occlusion should be minimized if there are two LADs supplying the same territory as a single LAD. However, in patients with long-standing atherosclerosis, it is possible that both LADs may be occluded. Therefore, it is pertinent to identify the coronary vessels accurately before certain interventions are taken. Our case proposes a novel variant (Type XIII) of the dual LAD anatomic phenomenon never before described in the world literature. Acknowledgement of this phenomenon can help guide accurate management in the future for patients with this variant.
